# Supplementary material for: Are carbon emissions trading and green financial instruments synergistic? -Comprehensive quantitative research based on content analysis
Source: PLoS One. 2024 Mar 7;19(3):e0298601. doi: 10.1371/journal.pone.0298601 (PMC10919601; doi:10.1371/journal.pone.0298601)
Supplement: S1 Data — (ZIP) [file pone.0298601.s001.zip › Data/地级市Z_F工作报告.zip!/╣π╬≈/▒▒║ú╩╨/└┤╫╘╣1⁄2╓┌║┼í╛┬φ┐╦╩2╛▌═°í┐_2013.docx]

各位代表：

现在，我代表北海市第十四届人民政府，向大会作政府工作报告，请予审议，并请市政协委员和其他列席人员提出意见。

一、实施北海三年跨越发展工程和2012年工作回顾

2012年是实施北海三年跨越发展工程的决战之年。在自治区党委、政府和市委的正确领导下，全市各族人民以邓小平理论、“三个代表”重要思想和科学发展观为指导，全面实施《广西北部湾经济区发展规划》，坚持“真抓实干、把事干成、造福百姓”的理念，按照“稳中求进、好中求快”和“稳增长、控物价、调结构、惠民生、抓改革、促和谐”的要求，以极大的信心、决战的勇气、十足的干劲，奋力夺取了北海三年跨越发展工程的全面胜利，推动北海进入了历史上最好的发展时期。

——经济发展实现大跨越。三年来，我们坚定不移地以加快发展为第一要务，加快转变经济发展方式，增强经济发展的内生活力和动力，综合实力大幅提升。2012年，全市地区生产总值突破600亿元、达到630.8亿元，比2008年翻一番以上，比上年增长21.8%；人均地区生产总值39425元，是2008年的2.23倍，首次超过全国平均水平；财政收入突破100亿元、达到100.09亿元，是2008年的3.7倍，比上年增长73.95%，成为广西第五个财政收入超百亿元的地市，人均财政收入位居全区前列；规模以上工业总产值突破1000亿元、达到1020.9亿元，是2008年的5.3倍，比上年增长69.5%，工业增加值达到245.4亿元、比上年增长59.5%；全社会固定资产投资突破700亿元、达到725.4亿元，是2008年的3.62倍，比上年增长20.3%。三次产业结构由2008年的25.5:34.9:39.6调整为20.3:49.1:30.6。

——优势产业实现大发展。三年来，我们坚定不移地走新型工业化道路，强力推进项目建设，努力打造三大千亿元产业，加快发展优势产业，产业支撑显著增强。2012年，电子信息、石油化工、临港新材料三大产业分别实现产值500亿元、300亿元和150亿元，分别是2008年的6.5倍、8.8倍和10.8倍，年均分别增长59.75%、72.39%和81.34%。北海工业园区、出口加工区产值相继超百亿元，铁山港（临海）工业区去年完成产值428亿元、跃居广西北部湾经济区14个重点产业园区第三位。中电产业园、中石化北海炼化、诚德新材料等一批重大项目建成投产，开创了北海项目引进建设的全新局面。

——开放合作迈出大步伐。三年来，我们坚定不移地实施开放带动战略，全面提高开放水平，扩大国内外交流合作。一批重大交通基础设施建成投入使用，亿吨大港建设扎实推进。先后获批为国家科技兴贸创新基地、加工贸易梯度转移重点承接地、外贸转型升级专业型示范基地。2012年，全市外贸进出口总额20.78亿美元，是2008年的2.92倍，电子信息产品和加工贸易产品进出口额位居全区第二。成功承办世界客属第24届恳亲大会，大型历史舞剧《碧海丝路》在国内外演出得到广泛好评，提升了北海的知名度和美誉度。

——城市建设管理获得大提升。三年来，我们坚定不移地打造生态宜居文明城市，坚持高起点规划、高标准建设、高效能管理，推动北海组团和铁山港组团互动发展，扎实推进城镇化，城市管理不断加强，城市功能逐步完善，城市品位明显提升。“七纵七横”城市主干路网基本形成，基础设施更趋完善，城镇化率提高至50.8%。森林覆盖率达36.24%，建成区绿化覆盖率达37.92%。先后获批为国家历史文化名城、国家园林城市。申办第四届广西园林园艺博览会喜获成功，参加广西第八届“南珠杯”竞赛获得市、县、镇3个特等奖，创建国家节水型城市通过考核，创建国家环保模范城市扎实推进。

——保障和改善民生取得大成效。三年来，我们坚定不移地坚持以人为本、造福百姓，加快社会事业发展，切实解决民生问题，人民生活水平不断提高。投入民生领域资金年均增长28.18%，累计达188亿元。如期完成为民办实事项目33大项共110件。实施三期民生路网工程，建设改造民生道路36条、总长31.5公里，有效解决市民出行难问题。实施千里乡村公路畅通工程，建设农村公路项目336个，惠及63万农村人口。2012年，全市社会消费品零售总额146.5亿元，是2008年的1.83倍，年均增长16.3%。城镇居民可支配收入21202元、增长13.6%，农村居民人均纯收入7227元、增长15.7％。

一年来，我们主要做了以下工作：

一是按照打造现代产业集聚基地的要求加快发展优势产业。北海炼化项目满负荷运行，年内完成产值282亿元，改扩建项目前期工作已启动；千万吨级炼化一体化、广西LNG及一批石化配套项目加快推进。电子信息产业聚集效应凸显，产值占全区三分之二。冠捷项目实现量产，生产显示器190万台；惠科显示器项目抓紧推进，首批配套项目已入驻投产；朗科、三诺项目分别建成5.2万和7.5万平方米厂房，实现试产或部分投产。诚德二期建成投产，一、二期共形成160万吨镍铬合金板坯的年产能，在国内排名前五位，三期金属压延工程投产试运行，四期250万吨/年冷轧工程项目征地拆迁已经完成。5000吨/天高蛋白饲料粕物流及加工项目建成投产，神华国华广投(北海)能源基地完成填海造地1100多亩，林浆纸一体化项目签订合作确认书，国投北海电厂二期等项目加快推进。北海出口加工区B区获国务院批准设立，封关建设工作全面展开，北海工业园区和高新区升级为国家级开发区工作扎实推进。工业集中度进一步提高，各产业园区完成总产值888亿元、占规模以上工业总产值的比重达87%。

二是按照推进统筹城乡发展的要求加快新农村建设。发放农作物良种补贴、成品油价格补贴等支农惠农政策资金13.46亿元。大力发展特色现代农业，建成高效设施农业基地30多个，辐射带动高效设施农业面积12.5万亩，农业设施化水平稳居全区首位，形成一批农产品名优品牌，建成一批农业科技示范基地、休闲农业示范点和农业标准化示范区。着力完善农业基础设施，完成农业固定资产投资25.86亿元。在全区率先实现100%行政村通硬底化公路目标，完成3200户农村危房改造任务，建成农村饮水安全工程32个、解决5.1万人的饮水安全问题。积极推进贫困地区基础设施建设和产业扶贫开发，启动新一轮社会扶贫工作，挂钩帮扶39个贫困村。成功抗击台风“启德”、“山神”，做到“船回港、人上岸”，各项损失降至最低。

三是按照建设生态宜居文明城市的要求全面推进城市规划建设管理。扎实开展“城市建设管理年”活动，开工建设城市基础设施项目40多个。启动《北海市城市总体规划》修编和廉州湾新区、冯家江新区等重要区域的规划编制，完成31个片区控制性详细规划修编。银滩大道等一批主干道路建设抓紧推进，新世纪大道、南珠大道、成都北路等一批城市道路进入竣工收尾阶段。积极实施合浦城市建设大会战。规划改造建设450条小街小巷，首批32条已完成。大力整治违法用地用海和违法建设行为，清理整治海域约21平方公里，拆除违章建筑60万平方米。理顺城市管理体制，深入实施“城乡清洁工程”和“绿满八桂”工程，大力整治交通秩序，完成主城区道路交通标志、标线、信号灯更新改造，道路机械化清扫率从15.7%提高至36%，公园绿地基本实现居住用地500米半径范围内全覆盖，荣获“创建全国无障碍建设先进城市”称号。加快污水截流管网建设和违法排污企业整治，开展侨港环境综合整治，加强银滩及近岸海域、涠洲岛生态环境整治与保护，涠洲岛珊瑚礁国家级海洋公园获批。全面完成年度节能减排任务，空气质量优良率达100%，在全国120个监测城市中名列第一，进一步树立了良好生态环境的城市品牌。

四是按照构筑旅游商贸物流中心的要求加快发展现代服务业。全年接待国内游客1311.2万人次、增长19.11%，国内、国际旅游收入达110.17亿元和3429.11万美元，增长28.01%和31.17%。涠洲岛旅游区开发建设掀起高潮，首批9个项目完成建设，二期15个项目抓紧推进。建成铁山港3、4号泊位，扎实开展5至10号泊位前期工作，基本完成石步岭三期5号泊位、邮轮码头水工部分建设。稳步推进钦北铁路扩能改造、玉林至铁山港铁路等交通项目，玉林至铁山港高速公路即将建成通车。完成机场换算旅客吞吐量76.19万人次，航线达到15条、通达城市15个；港口货物吞吐量1756万吨、增长10%，集装箱吞吐量8.52万标箱、增长20%。加快发展园区物流，努力降低企业物流成本。严格执行房地产市场各项调控政策，房地产市场健康平稳有序发展。狠抓金融生态环境建设，不良贷款率由7.86%下降至4.7%。

五是按照搭建开放合作重要平台的要求深化改革扩大开放。土地例行督察及土地矿产卫片执法检查顺利通过国家验收。完成9家市直国有企业改制，妥善安置职工2465人。着力培育推动企业上市，实现培育备案17家，完成申报发行1家，北生药业公司成功恢复上市，重组工作顺利推进。北海港口岸对外扩大开放获准列入国家“十二五”口岸发展规划，顺利完成审理阶段工作。成功承办第七届泛北论坛电子信息产业发展峰会，荣获2012百城论坛“友城战略发展奖”和“2012年广西国际友谊贡献奖”。新引进项目122个，实际到位资金354亿元、增长11.8%，实际利用外资2.57亿美元、增长18.25%。

六是按照保障和改善民生的要求全面加强社会建设。投入民生领域资金79亿元、增长22.93%，民生支出占财政支出的比重超过八成。年初承诺的各项为民办实事工程全面完成。建成村级公共服务中心88个、总数达到126个，提前三年实现农村书屋行政村全覆盖目标。海上丝绸之路项目列入中国世界文化遗产预备名单，提升了北海作为国家历史文化名城的影响力。大力发展文化产业，启动南珠博物馆等8个文化产业项目建设。荣获“全国文化体制改革工作先进地区”称号。高新技术企业新增5家、总数达23家，北部湾(北海)电子信息专业孵化器入驻企业56家，全市发明专利申请量达363件、增长2.8倍，与自治区科技厅建立厅市工作会商制度。编制完成全市中小学、幼儿园布局专项规划，新办海城区十七小，启动建设海城区十八小，结束了主城区超过10年没有新建公办中小学校的历史；合浦廉东小学和市机关幼儿园新园区交付使用，北海中学异地搬迁项目年内完成投资1.46亿元，市中职校暨职业教育中心新校区等项目加快建设。大力引进优质教育资源，北京八中北海分校开学招生，与北京师范大学合办北海附属中学扎实推进，创办桂林电子科技大学海洋信息工程学院获自治区教育厅批准。扎实推进引进两家全国知名医院工作，着力提高城乡居民公共卫生均等化服务水平，“新农保”实现制度全覆盖，“新农合”筹资水平提高到每人每年290元、参合率达98.18%，建成村卫生室335个，在基层医疗机构全面推行药品零差价销售，健康档案规范化电子建档109万人。推进农民体育健身工程，建成村级篮球场115个。积极做好人口计生工作，综合治理出生人口性别比问题成效显著。新开工保障性住房和棚户区改造住房7094套(户)，新增发放廉租住房租赁补贴959户，解决14483名城镇中低收入住房困难群众的居住问题。全市城镇新增就业4.15万人，城镇登记失业率3.1%；新增农村劳动力转移就业超过2万人。居民消费价格指数控制在2.6%。大力推进平安城市二期工程，抓好治安突出问题治理，有效化解一批矛盾纠纷和信访积案，信访总量明显下降。积极开展专项整治活动，全年无重大食品药品安全事故发生。深入排查整治安全生产隐患，各类安全事故下降6.67%。加快应急管理规范化建设，有效预防和处置突发公共事件，成功组织广西有史以来规模最大的海上搜救综合演练。其他各项事业也都取得了新进步。

各位代表!2012年是本届政府组成以来全面贯彻中央、自治区和市委的决策部署，全力推动北海跨越发展的一年，也是全面加强服务政府、责任政府、法治政府、廉洁政府建设，不断提高政府工作科学化水平的一年。

一年来，我们着力转变职能、改进作风。切实把政府工作的重点转到提供公共服务上来，做好政府该做的事。广泛聚集跨越发展的正能量，为干部搭好干事创业的舞台，努力创造让人民满意的业绩。切实加强干部作风建设，发扬“两脚泥”精神，真干实干苦干。年内开工重点项目101个、竣工68个、续建62个，完成投资188亿元。全面规范重要会议制度，召开政府常务会22次、土地(海洋)审批会10次、规委会17次，研究议题总数达566个，有力推动了各项工作。深入推进“扩权强县”和行政审批制度改革，44个部门向县区下放行政审批事项514项。面向全国招录事业单位人才，积极稳妥推进全市科长交流试点。加强政务督查，确保各项决策部署得到有效落实。

一年来，我们坚持敢于负责、为民谋利。切实维护社会稳定，为党的十八大胜利召开和北海跨越发展创造良好社会环境。敢于面对和妥善化解历史遗留问题，不断减轻发展包袱。坚持着眼长远发展和可持续发展，在项目引进、土地利用、资源开发、生态保护等方面多做造福子孙后代的事。着力破解“融资难、成本高”问题，积极利用社会资金建设公益项目，年内引进资金59.9亿元。在城区安排开发条件较好的1348亩储备土地用于医疗卫生、文化体育、中小学校等社会事业发展和公共设施建设，占全年土地供应总量的三分之一以上。努力解决群众反映强烈的热点难点问题，顺利实现上海路全线贯通。全面启动银滩等重要区域排污截流工程，推动成立海滩执法支队，实施建设项目海滩保护前置审批制度，加强全市范围内的岸线资源保护。

一年来，我们严格依法行政、规范运作。认真贯彻执行市人大及其常委会各项决议决定，主动向市人大及其常委会报告工作、向市政协通报情况，自觉接受人大及其常委会的法律监督和工作监督，自觉接受政协的民主监督，人大代表议案、建议和政协提案办结率100%。全面推行重大事项依法决策、科学决策、民主决策，实施的重大行政决策均经过合法性审查和市政府常务会议集体讨论决定。全面规范政府及其部门议事决策和执法、审批等行为，确保每一个具体行政行为都在法律和制度允许的范围内进行，年内发布29个重要工作规划，出台32个规范性文件。在全区率先成立行政执法监督局，进一步规范行政执法行为。着力解决行政处罚标准不一、宽严不同的问题，70%以上的部门已细化具体执行标准。

一年来，我们坚持廉洁从政、阳光行政。严格执行党风廉政建设责任制，深入开展重点领域渎职侵权专项整治。切实加强行政监督，扎实开展“审计整改年”活动，大力推行政府投资项目全程审计监督新模式，在全区率先运行公共资源交易电子招投标，将609项市本级行政审批事项纳入电子监察系统。将59个市直部门、公共服务单位651项事务纳入市政务服务中心集中办理和监管，平均日办结率73.55%，群众满意率达99.98%。全面推进政府信息公开，坚持市政府常务会议邀请人大代表、政协委员和监察、审计、法制部门负责人列席制度，切实保障人民群众的知情权和监督权；大力推动政务服务向基层延伸，全市90%的乡镇和街道建立了政务服务中心，市政府被评为广西政务服务工作先进单位、广西政务公开政府信息公开领先网站单位。

回顾一年来的工作，我们深刻体会到：一是必须坚持把科学发展观的普遍性要求与北海发展的具体实际紧密结合起来，贯彻到政府工作的全过程、各方面；二是必须坚持把发展作为解决北海一切问题的根本和关键，围绕跨越发展推进各项工作；三是必须坚持“真抓实干、把事干成、造福百姓”的理念，始终对人民负责、为百姓谋利；四是必须坚持走“产业带动跨越发展、文化引领持续繁荣、开放增添不竭动力”的发展路子，确保经济社会沿着正确的方向发展；五是必须坚持采取定人员、定职责、定时间、定进度的“四定”办法推进项目，充分发挥项目拉动经济社会发展的关键作用；六是必须坚持加强政府自身建设，不断提高推动发展、服务群众、促进和谐的能力和水平。

各位代表！一年来的成绩是在宏观经济形势错综复杂、各种不确定因素增多、维护社会稳定压力较大的情况下取得的，实属来之不易。这是自治区党委、政府和市委正确领导、科学决策的结果，是市人大及其常委会和市政协有效监督、大力支持的结果，是历届班子历任领导打下良好基础的结果，是全市人民共同奋斗的结果。在此，我谨代表市人民政府，向辛勤工作在各条战线上的干部群众，向驻市人民解放军、武警部队官兵，向各民主党派、工商联、人民团体和社会各界人士，向参与北海建设的投资者和创业者，向支持北海发展的港澳台同胞和海内外朋友，致以崇高的敬意和衷心的感谢！

我们清醒地看到，当前经济社会发展和政府工作还面临不少困难和挑战。一是宏观经济下行压力较大，给企业发展和项目建设带来了困难和挑战；二是铁山港口岸尚未开放和铁路尚未开通，制约了铁山港(临海)工业区建设和千亿元产业发展；三是城乡一体化发展步伐仍不够快，农民持续增收渠道不宽，以城带乡、以工促农的工作格局有待进一步完善；四是需要解决的关系群众切身利益问题仍然较多，保障和改善民生还有很多工作要做；五是少数政府工作人员服务意识、服务效率和执行能力有待进一步提高，廉洁从政工作还需进一步加强。对这些问题，我们一定高度重视，采取切实有效措施加以解决。

二、2013年的主要目标和任务

2013年是全面贯彻落实党的十八大精神的开局之年，是实施“十二五”规划承前启后的关键一年，是继续夯实北海在广西率先全面建成小康社会基础的重要一年，做好今年政府工作的意义十分重大。

今年政府工作的总体要求是：全面贯彻落实党的十八大及中央经济工作会议、自治区党委十届三次全会及全区经济工作会议和市委十届三次全会精神，以邓小平理论、“三个代表”重要思想、科学发展观为指导，坚持“真抓实干、把事干成、造福百姓”的理念，坚持“产业带动跨越发展、文化引领持续繁荣、开放增添不竭动力”的发展路子，巩固和扩大北海三年跨越发展工程成果，把握“稳中求进”的总基调，加大经济结构调整力度，发展壮大优势产业，统筹城乡一体化发展，努力深化改革开放，全面繁荣社会事业，切实保障和改善民生，深入推进生态文明建设，在新的起点上奋力开创北海科学发展新局面，为在广西率先全面建成小康社会打下坚实基础。

今年全市经济社会发展的主要预期目标是：地区生产总值增长13%、达到730亿元；财政收入增长10%、达到110亿元；全社会固定资产投资增长20%、达到870亿元；规模以上工业总产值增长27%、达到1300亿元，增加值增长18%；实际利用外资增长12%、达到2.8亿美元；外贸进出口总额增长15%、达到24亿美元；社会消费品零售总额增长15%、达到168亿元；城镇居民人均可支配收入增长14%、达到24100元；农民人均纯收入增长14%、达到8200元；居民消费价格指数控制在4%以内；全面完成自治区下达的节能减排控制性任务。力争实现地区生产总值增长16%、达到750亿元，财政收入增长12%、达到112亿元，全社会固定资产投资增长22%、达到885亿元，规模以上工业总产值增长42%、达到1450亿元，规模以上工业增加值增长20%，城镇居民可支配收入增长15%、达到24300元,农村居民人均纯收入增长15%、达到8300元。

为实现上述目标，我们将重点做好以下工作：

(一)推进结构调整优化，努力增创产业发展新优势。

坚持发展实体经济，加快打造三大千亿元产业，发展壮大战略性新兴产业，发展现代服务业、特色现代农业和海洋产业，壮大产业规模，形成产业集群。三次产业结构调整为19.2：49.3：31.5。

加快打造三大千亿元产业。完善三大千亿元产业园产业规划、控制性详细规划及各项专项规划。全力推进北海出口加工区B区建设，确保年内封关验收。加快园区基础设施建设，建成铁山港工业区排水明渠一期工程，开工建设中石化项目配套道路、铁山港航道疏浚二期扩建工程，完成中电北海产业园配套基础设施二期工程，加快推进三期、四期工程，开工建设三诺项目25万平方米标准厂房。继续引进一批大型电子企业和关联配套企业，加快惠科显示器项目建设，推动朗科、三诺等项目尽早投产达产，冠捷、长城能源等企业尽快完成增资扩产。加快北海炼化项目扩能改造，完成混芳精馏项目和生产航煤技术改造，积极推进二期千万吨级炼化一体化项目。建成广西LNG项目引堤和陆域形成工程，加快接收站、输气管道和码头建设。加快和源化工、中航化工、新鑫碳四等项目建设，延伸石化产业链。推动诚德三期工程全面投产，开工建设扩能改造项目和四期冷轧工程120万吨/年生产线，引进一批下游企业落户新材料产业园。力争北海工业园区、北海高新区成功升级，加快合浦工业园扩容和云南临海产业园建设，支持铁山港东岸临海产业园申报广西北部湾经济区重点园区。力争电子信息、石油化工、临港新材料三大产业分别完成产值580亿元、320亿元和200亿元。

加快发展以旅游为龙头的现代服务业。推进银滩景区提升工程，加快打造国际滨海旅游度假区。推进涠洲岛整体开发，抓紧完成风景名胜区规划和旅游产品等专项规划编制，加强生态和资源保护，加快二期15个项目建设，开工建设涠洲岛环岛路、回建区、度假酒店等项目。抓好涠洲岛鳄鱼山景区创5A和金海湾红树林生态休闲旅游区创4A等景区标准化建设，加强北海老城保护和开发利用。鼓励亲海度假酒店建设，建成富丽华等高星级酒店，促进休闲度假和商务旅游业发展。加快发展滨海旅游业，抓紧完成北海至越南海上旅游航线复航工作。按照建立现代企业制度的要求，打造市旅游集团等旅游龙头企业，并积极推进改制上市。加强和改进旅游市场监管，严厉打击非法“海上游”和以次充好等行为，提高旅游服务质量和规范化水平。全年接待国内游客1500万人次，国内旅游收入达到130亿元。加快交通物流基础设施建设，推动形成互联互通的国际大通道。建成南宁至北海高速铁路、玉林至铁山港铁路、铁山港铁路支线、中石化铁路专用线，开工建设铁山港1-4号泊位铁路专用线，力争开工建设合浦至湛江铁路；开通玉林至铁山港高速公路，力争开工建设贵港至合浦高速公路、广西滨海公路山口经沙田至红树林支线、高速公路合浦收费站至北铁公路段改造、北铁大道改造工程；开工建设铁山港5至6号泊位和中石化液体化工码头，加快石头埠作业区1号、2号泊位建设，建成北海邮轮码头。培育北海至香港集装箱定期班轮航线，扶持物流企业发展，降低企业物流成本。继续培育扶持航空市场。结合实际落实宏观调控政策，合理适度发展房地产业。继续大力支持金融系统开展强化管理、强化服务的“双强化”活动，进一步优化金融生态环境。积极发展会展、通信等服务业，提升第三产业比重和水平，优化产业结构。

加快发展特色现代农业。全面落实农作物良种补贴、成品油价格补贴等各项扶持政策，调动农民发展生产积极性，保持农民收入持续较快增长。稳定粮、糖、油和水果、蔬菜等主要农产品生产，推进“万元增收”工程，抓好设施农业基地、菜篮子产品生产基地和休闲农业基地建设，扶持大棚种植和无公害蔬菜生产，推进农业生产标准化建设，提升特色农业现代化水平。打造一批年销售收入超5亿元和超亿元的龙头企业、骨干企业，发展一批农民合作经济组织和农产品行业协会，加快农业产业化进程。加大农业投入品监管和农产品质量检测力度，提高农产品安全水平。大力发展林下经济，培育林下种养走廊和金花茶、罗汉松种植品牌。推动特色畜牧养殖业规模化发展。

加快发展海洋产业。着力提升海洋传统产业，培育壮大海洋新兴产业，加大海洋基础建设投入，提高海洋资源开发和管控能力，建设海洋强市。在发展临海工业、港口物流业、滨海旅游业的同时，加快发展其他海洋产业。推进北海内港、营盘中心渔港、沙田渔港、西场大风江渔港建设，逐步解决港窄船多的问题。完成北海国家农业科技园区建设，争取通过国家验收，积极筹划建设北海海洋产业科技园。积极开发南沙渔场，稳步发展远洋渔业，大力发展优势水产养殖业，促进水产品精深加工出口。规划建设珍珠养殖永久性基地和南珠主题公园，完善国家珍珠质检中心建设，制定南珠产业标准，加快南珠产业发展，提升南珠品牌形象。推进海洋渔船更新改造项目，提升装备技术水平。

加快培育壮大其他优势产业。促进修造船、建材、机械等产业升级转型，积极发展先进装备制造、海洋生物和新能源产业，加快华润水泥等项目建设。推进神华能源基地、国投北海电厂二期和华电合浦电厂一期工程，打造北部湾能源基地。建设500千伏北海输变电、220千伏久铁输变电和神华能源基地配套送出工程，彻底解决北海网架薄弱问题。力争开工建设林浆纸一体化项目。加快发展农产品加工业，完成产值180亿元。建设北海高新区软件和信息服务产业基地、中国科技开发院高技术服务业北海基地、国家级科技企业孵化器、软件与集成电路促进中心北部湾平台，推动信息化和工业化深度融合。

(二)推进统筹城乡发展，加快社会主义新农村建设。

围绕在全区率先基本形成城乡一体化发展格局的目标，加大新农村试点和建设力度，加快城乡一体化步伐，增强农村发展活力，逐步缩小城乡差距，促进城乡共同繁荣。

加快农村基本公共服务设施建设。加大城镇基础设施向农村延伸力度。加快城乡公路网规划修编和调整，完善干线公路网、聚集区和中心村路网。加快江河治理、海河堤标准化建设、病险水库除险加固、灌区节水改造、重点水源建设、农田水利建设、水资源保护和水土保持、水能资源开发、城镇防洪排涝整治、水利信息化建设等骨干水利项目建设。推进千里乡村公路畅通工程。建立农村供水社会化服务体系，逐步实现城乡供水一体化。推进水库库区、移民安置区、国有林场垦区危旧房改造。继续实施水库移民新村建设工程。完善城乡一体的供电网络，逐步实现同网同价。继续加大扶贫攻坚力度，为在全区率先完成新一轮扶贫攻坚任务奠定坚实基础。

加快农村生态环境建设。加大小流域综合治理力度，加快建设高效的农业生产生态防护体系、城乡宜居的森林生态环境体系、持续稳定的生态安全体系，逐步形成城乡绿化一体化格局。推进农村环境连片整治，加强饮用水源地环境综合整治和农业面源污染防治，推进国家级生态乡镇建设。加快农村能源建设，推广沼气、秸秆利用等生物质能及小水电、风能、太阳能等清洁和可再生利用技术。实施农村清洁工程，加快改水、改厨、改厕、改圈步伐，推进垃圾集中处理，不断改善农村卫生条件。

加快健全农村社会化服务体系。加强农业公共服务能力建设，建立健全农业技术推广、动植物疫病防控、农产品质量监管等公共性服务机构，逐步建立村级服务站点。加强农村信息网络和通信基础设施建设，提高农村通信覆盖率、电话普及率、行政村通宽带率。加快“万村千乡”市场工程建设。支持农业服务业发展，为农户提供产供销一体化服务，着力破解农产品销售瓶颈。实施农村实用科技人才培养工程。推进义务教育均衡发展，促进教育质量一体化、师资配备均衡化、基础设施标准化。健全农村卫生服务体系，将公办村卫生室全部纳入基本药物制度实施范围。落实农村食品安全监管“一专三员”机制，提高农村食品安全水平。

(三)推进新型城镇化，全面建设美丽北海。

推动工业化和城镇化互动发展、城镇化和农业现代化相互协调，加快城镇化步伐，提高城镇化质量，积极参与南北钦防同城化。深入开展“城市建设管理年”活动，积极创建国家环保模范城市、国家森林城市、国家生态文明城市和中国人居城市，推进人与自然和谐发展，力争在生态文明建设方面走在全区前列。

强化规划引导和土地节约集约利用。坚持“规划一支笔、建设一张图、管理一盘棋”，推进城乡规划全覆盖，加快城市总体规划修编和交通综合规划、市政规划、村镇规划、蓝线和绿线规划、廉州湾沿岸开发建设总体规划等重要规划编制。加强城市特色研究，严把规划审批关，构筑独具滨海特色的城市风貌。发挥辖区政府积极性，建立长效机制，严厉打击城乡违法用地用海和违法建设行为。做好土地矿产卫片执法检查，确保实现“零约谈、零问责”目标。积极盘活存量土地，确保耕地占补平衡，有效保障重点产业项目和公益设施、民生项目用地用海。加强项目用地用海投资强度审核，推动工业向园区集中，提高土地利用效益。

高标准高水平推进新城区建设。重点抓好廉州湾新区、冯家江新区、银滩海岸沿线、涠洲岛等新城区建设。结合北海工业园区和合浦工业园的发展，加快廉州湾新区规划建设，推动北海大道延长线与合浦大道连接建设，支持合浦县城建设大会战，促进合浦县城与市区一体化发展，加快北海组团建设。结合第四届广西园林园艺博览会园区建设，加快冯家江新区开发，全面启动基础设施建设，力争开工建设城市规划(博物)馆、广电中心等项目。结合银滩改造建设和涠洲岛整体开发，加快新城区规划建设。积极推进铁山港滨江生活区等新城区建设。抓好银滩大道、金海岸大道等重要道路建设，争取做到修通一条道路、带动一片区域开发。注重改善新城区生态环境，同步建设市政配套和公共服务设施，实现开发一片、完善一片。积极开展城镇化村镇试点，加快中心城镇建设，提升产业发展、公共服务、吸纳就业、人口集聚功能，有序推进农业转移人口市民化，城镇化率提高到51%。

不断提高城市建设管理水平。加强城市基础设施建设，开工建设红坎污水处理厂二级处理二期工程，完成广东南路、侨港区域、银滩中路污水截流工程，加快重庆路、南京路、江苏路、浙江路等市政道路建设。开工建设内涝整治二期工程，提高防汛排涝抢险能力，逐步解决城区内涝问题。推进小街小巷改造工程，完成主城区168条小街小巷建设。继续推进重要节点风貌整治。深入开展城乡清洁工程和净化、绿化、彩化、亮化、美化工程，积极筹备第四届广西园林园艺博览会。继续完善城市保洁设施，机械化清扫率提高到40%以上。扩大市场化保洁范围，推进市政设施市场化维护和管理。完善路灯监控系统,路灯设施完好率达到95%以上。开展主城区地下管线普查，建立完善规划管理数据库。加大交通执法和疏导力度，提高城区道路通畅水平。改进城市管理行政综合执法，坚持疏堵结合，整合资源，改进机制，提高效率，推行人性化管理。依法保护并合理改造老城区，抓好“城中村”和侨港改造建设，不断改善人居环境。

加大自然生态系统和环境保护力度。推进“绿满八桂”、“北部湾绿色屏障”建设。加强空气质量监测，做好PM2.5实时监测和发布工作。加强海洋生态保护，实施近岸海域、海岛及海洋生态综合整治工程，重点抓好入海排污口、市区北岸、涠洲岛西南海岸带的综合整治，启动建设涠洲岛珊瑚礁国家级海洋公园，创建国家级海洋生态文明示范区。强化水源地保护，加强地下水污染防治和开采控制，加大南流江等重点河流水污染防治力度。大力发展生态产业和循环经济，全面落实节能减排措施。加快城乡垃圾、污水处理设施建设，开工建设白水塘生活垃圾焚烧项目。逐步建立完善流域水资源、近海资源等各类资源有偿使用的生态环境保护补偿制度。加大环保执法力度，深入开展整治违法排污企业专项行动，切实保障群众健康。

(四)拓展改革开放广度深度，积聚加快发展的动力和能量。

推进重点领域和关键环节改革，加快构建充满活力、富有效率、更加开放的体制机制。实施更加积极主动的开放战略，加快构建全方位、多层次、宽领域的对外开放新格局，增强发展活力。

加快推进各项改革。积极发展非公有制经济，最大限度取消民间投资限制，鼓励民间资本进入市政、金融、能源、教育、医疗等领域。深入实施“抓大壮小扶微”工程，落实各项鼓励扶持措施，培育发展一批中小企业和微型企业。积极引进各类金融机构，稳步发展小额贷款公司，培育面向中小企业、微型企业和“三农”的金融机构。建立完善激励与约束并重的财税政策。加强国有资产运营监管。优化支出结构，重点向农村、社会事业发展的薄弱环节倾斜，向困难地区、基层、群众倾斜。推进户籍制度改革，放宽城镇落户条件。坚持依法自愿有偿原则，引导农村土地承包经营权有序流转，鼓励和支持承包土地向专业大户、家庭农场、农民合作社流转。深化集体和国有林权制度改革，启动集体林权制度配套改革。推进水利改革，加快形成以公共财政投入为主的政府主导、市场补充、群众参与的水利投融资体制。推进城市管理体制改革，加快形成条块结合、重心下移、统筹协调、分工合理的城市管理新格局。推进科技、教育、文化、医疗卫生等体制改革和干部人事制度、事业单位改革。

进一步扩大开放合作。着力提高招商引资水平，制定更加优惠政策，采取更加有效措施，营造更加浓厚氛围，创新招商引资方式方法，突出重点、拓宽领域、注重实效，以大招商引进大项目，以大项目促进大发展。积极推进泛北部湾区域合作，进一步扩大与东盟国家的交往与合作。深化与珠三角、港澳台、西南地区等区域在交通、能源、旅游、教育、农业等领域的务实合作，发挥比较优势，扩大承接产业转移成效。积极推进和深化与央企的合作。加快建设国家科技兴贸创新基地、加工贸易梯度转移重点承接地、国家外贸转型专业型示范基地（水产品），促进外贸结构优化和产业升级。积极申报国家电子商务示范基地。大力推进口岸基础设施建设，力争北海港口岸扩大开放获得国务院批准。

(五)繁荣发展社会事业，提高协调发展水平。

坚持改革发展成果与人民共享，推动经济社会协调发展。继续加大投入，促进各项社会事业全面进步。积极引进和培育优质社会事业资源，提升城市宜居水平，满足人民群众日益增长的公共服务和精神文化需求。

大力繁荣发展先进文化。充分发挥北海国家历史文化名城优势，完善文化基础设施，加快构建公共文化服务体系，力争开工建设市群艺馆迁建、合浦汉文化考古遗址公园等项目。发展特色文化产业，规划建设文化产业园区和街区，打造海洋文化、合浦汉文化、珍珠贝雕、水彩画等品牌。推进联合申报“海上丝绸之路”世界文化遗产。加强文物和非物质文化遗产的保护利用，加强历史文化名城、名镇、名村保护。不断繁荣文化创作，深入实施艺术精品工程。广泛开展群众文化活动。进一步拓宽对外文化交流合作的范围和领域，积极推动北海文化“走出去”。加强文化市场管理。把握正确导向，做好广播电视宣传和新闻出版工作，抓好网络媒体建设和管理。

坚持教育优先发展。全面实施素质教育，加强教师队伍建设。下大力气解决中小学校布局不合理、“入园难、入学难”和“大班额”问题，全面实施中小学、幼儿园专项布局规划，完成北海中学异地搬迁，加快建设海城区十八小、银海区一小，开工建设北海十中、北师大附中和银海区二小，积极推进中小学校危旧房改造。大力发展现代职业教育，完成市中职校迁建，加快北海职业学院搬迁。积极发展高等教育，加快引进两所高校到北海办学，创办桂电海洋信息工程学院，力争北海艺术设计职业学院通过升级本科评估。完善困难学生资助体系，提高资助水平。

切实提高医疗服务水平。深化医药卫生体制改革，健全覆盖城乡的医疗卫生服务网络，巩固完善国家基本药物制度和新农合制度，大力推行重大疾病医疗保障工作，推进县级公立医院综合改革，加强基层卫生队伍建设。稳步推进基本公共卫生服务逐步均等化，建立健全突发公共事件卫生应急体系。加强医疗卫生基础设施建设，加快引进两家全国著名医院，建成银海区人民医院，加快市第二人民医院迁建，建设市全科医生培训基地，启动市人民医院异地扩建和广西海难紧急医学救援中心建设，提升整体医疗服务能力，扎实推进全国卫生城市创建工作。深入开展医患纠纷人民调解工作，积极构建和谐医患关系。广泛开展爱国卫生运动，促进人民身心健康。

加快发展其他社会事业。实施创新驱动发展战略，完善科技创新体系。大力提升重点企业自主创新能力，推进高新技术产业发展。加强知识产权保护，促进发明创造，力争发明专利申请量突破500件。大力加强四级基本公共体育设施建设，推进北部湾体育中心一期、市体育馆、山东省体育局北海训练基地、北海综合健身馆项目。深入开展全民健身运动，不断提高竞技体育水平。坚持计划生育基本国策，继续稳定低生育水平，提高出生人口素质，加强流动人口计生服务管理。启动建设北海市防震减灾科普教育基地，增强公众防震减灾意识。推动档案、地方志、人民防空等其他各项社会事业加快发展。

(六)切实保障和改善民生，提升人民幸福感和满意度。

坚持以不断提高群众生活水平和质量为目标，实施城乡居民收入倍增计划，进一步加强社会建设，多谋民生之利、多解民生之忧、多办民生实事，努力让全市人民过上更加美好的生活。

积极促进就业创业。优化就业结构，大力开发第三产业就业渠道，增加就业比重。统筹城乡就业，切实做好高校毕业生、城镇就业困难人员、农村转移劳动力、失地失海农民、转产转业渔民、退役军人等重点群体的就业工作。加强职业技能、岗位技能提升培训，促进充分就业。实施全民创业工程，完善和落实小额担保贷款财政贴息、税费减免等鼓励政策，优化创业环境，促进各类群体自主创业，以创业带动就业。完善覆盖城乡的公共就业服务体系,城镇登记失业率控制在4%以内。

统筹推进城乡社会保障体系建设。健全完善城乡居民低保、养老保险、社会救助和被征地农民社会保障制度，全面建成覆盖城乡的社会保障体系。整合城乡居民基本养老、医疗保险制度，确保基本养老金社会化发放率保持100%。完善基本医疗保险市级统筹制度，逐步实现“一卡通”就医即时结算。完善社会救助体系，提升社会救助水平。发展慈善事业，做好优抚、老龄、残疾人等工作。健全社会福利制度，加快儿童福利机构建设，保障妇女儿童合法权益。扩大保障性住房建设，加快农村危房改造，为人民群众创造更舒适的居住环境。

加强和创新社会管理。大力营造和谐稳定的社会环境、公平竞争的市场环境、规范有序的法制环境、安居乐业的生活环境。加强社会主义精神文明建设，抓好第六轮自治区“文明城市”创建工作。深入推进普法和依法治理，争创全国法治城市。完善矛盾纠纷排查调处和信访工作制度。落实领导干部安全生产“一岗双责”和重大隐患五级监管治理制度，强化食品药品等公共安全体系和企业安全生产基础建设，遏制烟花爆竹、交通运输、渔港安全、非煤矿山、危险化学品等重特大事故。全面推进平安城市二期工程，力争在全区率先实现社会治安监控和智能交通系统主城区全覆盖。加快完善立体化社会治安防控体系，依法严厉打击“两抢一盗”、非法传销、圈海霸海等违法犯罪活动，保障人民生命财产安全。健全统一指挥、反应灵敏、协调有序、运转高效的应急管理机制，扎实推进应急管理规范化建设，有效预防和处置各类重大突发事件和公共危机。

大力实施为民办实事工程。筹措资金约13.2亿元，办好以下10件工程：

1．社保惠民工程。筹措资金4276万元，将城市低保和农村低保标准分别提高到每人每月350元、每人每年2000元。筹措资金1500万元，实现城镇新增就业2.5万人、新增农村劳动力转移就业1.2万人。

2．卫生惠民工程。筹措资金约3.79亿元，将新型农村合作医疗筹资标准提高到每人每年340元，财政对新型农村合作医疗补助提高到每人每年280元。筹措资金约900万元，组建北海市120紧急救援中心，完善120急救中心调度指挥系统和急救分中心基础设施建设，增配3台监护型救护车及车载设备，配备远程医疗急救会诊系统。

3．教育惠民工程。筹措资金7587万元，新建县区、乡镇公办幼儿园10所，改制1所，改建扩建2所，基本解决入园难问题。筹措补助资金2160万元，资助1.8万名农村义务教育阶段家庭经济困难寄宿生生活费。筹措资金1000万元，为全市约5000名中等职业学校在校生免除学费。

4．强基惠民工程。筹措资金6000万元，建设农村公路约100公里。筹措资金689.9万元，为40个行政村修建农村屯级道路25.05公里，惠及5253户家庭共2.37万人。筹措资金2994.87万元，解决3.48万农村居民饮水安全问题。筹措资金1000万元，建设水库移民新村20个。

5．安居惠民工程。筹措资金5.39亿元，新建公共租赁住房3068套、经济适用住房150套、廉租住房436套，新增租赁补贴发放400户。筹措资金6400万元，改造农村危房3200户。

6．城建惠民工程。筹措资金645万元，建设城市震害预测与管理信息系统，改造北部湾东路垃圾转运站，新建、改建公共厕所10座，安装果皮箱垃圾桶300个。

7．农补惠民工程。筹措资金1020万元，实现水稻、玉米等粮食作物良种补贴全覆盖。筹措资金1200万元，发放农业机械购置补贴。筹措资金100万元，建设“放心粮油店”10个、“放心农资店”5个、“日用品农家店”5个。

8．生态惠民工程。筹措资金295万元，实施“绿满八桂”绿化造林工程，绿化村屯15个，送珍贵树种下乡50万株。筹措资金45.4万元，实施森林生态效益补偿项目，管护生态公益林9.09万亩。筹措资金53万元，购置监测仪器，加强饮用水水质监测，每月对市区6处集中式饮用水源进行23项常规分析，每年进行一次39项水质全分析。筹措资金1371万元，对市区北岸半岛公园至高德港段约2公里海岸带实施综合整治。

9．文体惠民工程。筹措资金684万元，建设18个村级公共服务中心，总建筑面积2880平方米。筹措资金30万元，开展文化惠民演出100场次。筹措资金82.32万元，补贴放映农村公益电影4116场，实现全市每个行政村每月放映一场电影。筹措资金120万元，安装20条健身路径。

10．计生惠民工程。筹措资金192万元，为8000对符合政策的计划怀孕夫妇进行免费孕前优生健康检查。

这10件实事工程，件件事关发展、事关民生、事关社会和谐稳定。我们一定要把实事办实办好，让人民群众共享改革发展的成果，感受到党和政府的温暖！

三、努力建设人民满意的政府

各位代表！当前北海已经站在新的起点上，新形势、新任务对政府建设提出了更高的要求。我们将坚持“真抓实干、把事干成、造福百姓”的理念，深入推进服务政府、责任政府、法治政府、廉洁政府建设，更好地推动北海科学发展、跨越发展。

我们将更加注重改进作风，提升为民谋利能力。坚决落实中央“八项规定”，以让人民群众满意为最高标准，用踏石留印、抓铁有痕的劲头狠抓作风建设。力戒空谈，崇尚实干，不当旁观者、不做评论员，真抓实干、干出实效。切实改进会风文风，坚持“短实新”、反对“假长空”，开解决实际问题的会，说群众听得懂的话，行简明扼要的文。继续改进调查研究，轻车简从、深入基层，掌握实情、解决问题。坚持密切联系群众，改进和提高群众工作水平，真心实意为群众排忧解难。对重大民生事项实行承诺制，做到言必行、行必果，以转变作风的新成效取信于民。

我们将更加注重转变职能，提升服务发展能力。加强和改进经济社会事务管理，创新公共服务体制机制，提高公共服务质量，努力为各类经济主体创造公平、良好的发展环境。深化行政体制改革，加快形成权责一致、分工合理、决策科学、执行顺畅、监督有力的行政管理体制。巩固扩权强县成果，继续深化审批制度改革，做到能减则减、能放则放、能快则快、能优则优。加强学习型政府建设，提高把握经济规律、破解发展难题、推动科学发展的能力和水平。优化政务服务，推进政务服务规范化和均等化。加强政务督查，推进电子政务建设，加强行政效能电子监察。强化行政问责，切实提高政府执行力，确保政令畅通、令行禁止。

我们将更加注重法治建设，提升依法行政能力。加强对权力运行的制约和监督，切实做到“把权力关进制度的笼子”，不断提高政府工作规范化水平。逐步完善施政行为公开机制，推进依法决策、科学决策、民主决策。深入开展依法行政示范点创建活动，强化行政执法监督，公正文明规范执法。建立完善行政争议预防和化解机制。自觉接受人大及其常委会的依法监督和政协的民主监督，高质量做好人大代表建议和政协提案办理工作。认真听取各民主党派、工商联、无党派人士和人民团体的意见建议，主动接受公众监督和媒体监督。推进政务公开，完善政府信息发布制度和平台，让权力在阳光下运行。

我们将更加注重廉政建设，坚持勤俭办事。坚持标本兼治、综合治理、惩防并举、注重预防方针，深入推进惩治和预防腐败体系建设。严格执行党风廉政建设责任制和廉政准则，落实领导干部重大事项报告制度。加强反腐倡廉教育和廉政文化建设，坚决反对特权思想、特权现象。始终保持惩治腐败的高压态势，坚持“老虎”、“苍蝇”一起打，既坚决查处领导干部违纪违法案件，又切实解决发生在群众身边的不正之风和腐败问题。大力弘扬节约光荣、浪费可耻观念，厉行勤俭节约，反对铺张浪费。严格控制“三公”经费支出，把有限的资金用在推动发展、改善民生、增进群众福祉上。全体政府工作人员特别是领导干部一定要以身作则、率先垂范，做到干部清正、政府清廉、政治清明，进一步形成风清气正的良好局面。

各位代表!回顾过去，我们团结奋进，取得显著成就；展望未来，我们任重道远，仍须不懈努力。让我们紧密团结在以习近平同志为总书记的党中央周围，在自治区党委、政府和市委的正确领导下，凝聚全市人民的智慧和力量，众志成城、奋力拼搏，攻坚克难、扎实工作，为开创北海科学发展新局面、在广西率先全面建成小康社会而努力奋斗！
